# Diurnal Salivary Cortisol in Sarcopenic Postmenopausal Women: The OsteoLaus Cohort

**DOI:** 10.1007/s00223-021-00863-y

**Published:** 2021-05-18

**Authors:** Elena Gonzalez Rodriguez, Pedro Marques-Vidal, Bérengère Aubry-Rozier, Georgios Papadakis, Martin Preisig, Christine Kuehner, Peter Vollenweider, Gerard Waeber, Didier Hans, Olivier Lamy

**Affiliations:** 1 Interdisciplinary Center for Bone Diseases, Service of Rhumatology, Lausanne University Hospital and University of Lausanne, Lausanne, Switzerland; 2 Service of Internal Medicine, Department of Medicine, Lausanne University Hospital and University of Lausanne, Lausanne, Switzerland; 3 Service of Genetic Medicine, Department of Medicine, Lausanne University Hospital and University of Lausanne, Lausanne, Switzerland; 4 Service of Endocrinology, Diabetology and Metabolism, Department of Medicine, Lausanne University Hospital and University of Lausanne, Lausanne, Switzerland; 5 Epidemiology and Psychopathology Research Unit, Department of Psychiatrics, Lausanne University Hospital and University of Lausanne, Lausanne, Switzerland; 6 Research Group Longitudinal and Intervention Research, Department of Psychiatry and Psychotherapy, Central Institute of Mental Health, Medical Faculty Mannheim, Heidelberg University, Heidelberg, Germany; 7 Interdisciplinary Center of Bone Diseases, Service of Rheumatology, CHUV, Lausanne University Hospital, Rue Pierre-Decker 4, 1011 Lausanne, Switzerland

**Keywords:** Salivary cortisol, Sarcopenia, Body composition, OsteoLaus, Cohort

## Abstract

Sarcopenia, similar to hypercortisolism, is characterized by loss of muscle mass and strength. Cortisol circadian rhythm changes with aging (blunted late-day nadir values) were suggested to contribute to this decline. We aimed to explore the relationship between diurnal salivary cortisol values and sarcopenia diagnosis and its components in postmenopausal women. This is a cross-sectional study within the OsteoLaus population-based cohort in Lausanne (Switzerland). Participants had a body composition assessment by dual X-ray absorptiometry (DXA), a grip strength (GS) measure, and salivary cortisol measures (at awakening, 30 min thereafter, 11 AM (sc-11AM) and 8 PM (sc-8PM)). Associations between salivary cortisol and sarcopenia diagnosed by six different criteria (based on appendicular lean mass (ALM) assessed by DXA, and muscle strength by GS), and its components, were analyzed. 471 women aged > 50 years (63.0 ± 7.5) were included. Various definitions identified different participants as sarcopenic, who consistently presented higher salivary cortisol at 11 AM and/or 8 PM. There were no associations between salivary cortisol levels and ALM measures, either absolute or after correction to height squared (ALM index) or body mass index. GS was inversely correlated to sc-11AM (*r* = − 0.153, *p* < 0.001) and sc-8PM (*r* = − 0.118, *p* = 0.002). Each 10 nmol/l increase of sc-11AM, respectively sc-8PM, was associated with a GS decrease of 1.758 (SE 0.472) kg, respectively 2.929 (SE 1.115) kg. In postmenopausal women, sarcopenia is associated with higher salivary cortisol levels at 11 AM and 8 PM. An increase of daily free cortisol levels in the physiological range could participate to sarcopenia development by decreasing muscle function in postmenopausal women.

**Supplementary Information:**

The online version contains supplementary material available at 10.1007/s00223-021-00863-y.

## Introduction

Sarcopenia, characterized by the loss of skeletal muscle mass and strength, is a major risk factor for physical frailty, poor health-related quality of life, and premature death in older people [1], as well as a negative prognostic factor in numerous diseases [2]. Its prevalence ranges from 1 to 29% in community-dwelling populations [3]. Different definitions of sarcopenia have been proposed using different methods or cut-off points for assessing muscle mass and function [4], which lead to very large differences in sarcopenia prevalence in a given population [5] and even classify different individuals as *sarcopenic* [6]. Many different complex and not yet fully understood pathophysiological mechanisms may be implicated in its development [7].

Overt hypercortisolism leads to muscle atrophy and weakness [8]. With aging, changes in the cortisol circadian rhythm induce long-term exposure to mildly higher cortisol levels, due to higher late-day and evening nadir values [9, 10]. These can be observed in salivary cortisol, an easy and non-stressful method of cortisol measurement [11]. It has higher clinical utility, because of the better convenience than other sampling methods (can be performed in the outpatient setting), and because it represents unbound active cortisol levels.

Modifications of the hypothalamic-pituitary-adrenocortical axis with aging have been suggested to contribute to the decline in lean body mass [12], although data are scarce and heterogeneous. Patients with mild hypercortisolism in the context of an adrenal incidentaloma have either lower lean mass as measured by bioimpedance [13] or lower body mass index (BMI)-adjusted lean mass assessed by dual-X-ray absorptiometry (DXA) [14]. Previous studies have suggested that higher diurnal or evening salivary cortisol, or blunted cortisol circadian rhythm, are associated with low muscle function measured by different methods [15–18]. Only one small study (*n* = 45) [19] indicated a relationship between sarcopenia diagnosis and blunted diurnal variation of cortisol.

In this context, we aimed to determine whether cortisol levels at different time-points, or the area under the curve, were associated with sarcopenia, taking into account definitions including different diagnostic cut-offs for lean mass by DXA, with or without muscle function measured by peak grip strength (GS).

## Materials and Methods

### Setting

We analyzed data from the previously described OsteoLaus sub-study of the CoLaus|PsyCoLaus study [20]. Briefly, CoLaus|PsyCoLaus is an ongoing prospective cohort study on cardiovascular and mental diseases determinants in a population-based sample of 6738 randomly selected individuals from Lausanne, Switzerland [21]. The OsteoLaus sub-study aims to compare different models of fracture risk prediction and to assess the relationship between osteoporosis and cardiovascular diseases [22]. OsteoLaus invited all women aged 50–80 years within 6 months of first follow-up of the CoLaus|PsyCoLaus study (September 2009–December 2012) to have a DXA scan; 85% accepted [22]. Also, at first follow-up CoLaus|PsyCoLaus participants aged > 50 benefited of GS measures in the context of a frailty sub-study, and of an assessment of the corticotropic axis by measures of salivary cortisol circadian rhythm [23] (Supplementary Fig. 1).

The institutional Ethics Committee of the University of Lausanne, later the Ethics Commission of Canton Vaud (www.cer-vd.ch), approved the baseline and first follow-up of CoLaus study (references 16/03 and 33/09), and the OsteoLaus study (reference 215-09). All participants signed a written informed consent after having received a detailed description of the goal and funding of the studies. Human studies are in accordance with the ethical standards of the 1964 Declaration of Helsinki and its later amendments.

### Participants

All participants were included except if they presented the previously defined exclusion criteria [20] or lacked data. Briefly, participants were excluded if they did not participate to the first CoLaus|PsyColaus follow-up, had no salivary cortisol measure, or lacked grip strength (GS) measurement, as well as if they were current smokers or treated with systemic glucocorticoids ≥  3 months. As in our previous study [20], salivary cortisol values > 4 standard deviations (SD) higher than the mean were considered as outlier values and excluded.

### Salivary Cortisol Measures

At the end of the interview of the first psychiatric follow-up assessment [24] participants received little swabs (Salivettes, Sarstedt, Sevelen, Switzerland) for salivary cortisol collection on a working day at awakening, 30 min thereafter, at 11 AM (sc-11AM), and at 8 PM (sc-8PM). Subjects were instructed not to brush their teeth and to refrain from eating, drinking, smoking, and exercising 30 min prior to and during the sampling procedure [25]. Saliva samples were stored at patient’s freezers and then at − 20 °C at the laboratory until biochemical analysis. Free cortisol levels were measured using a commercially available chemiluminescence assay (IBL, Hamburg, Germany). Inter- and intra-assay coefficients of variability were <  9%.

Diurnal Area Under the Curve values (sc-AUC) were calculated using the *AUCground* trapezoid method as performed by Pruessner et al. [26], and Cortisol Awakening Response (CAR) by subtracting the salivary cortisol awakening value from the 30 min after awakening value.

### Anthropometric Measures and Body Composition Assessment

All participants had their height measured using the same portable stadiometer (Seca version 216, Seca, Chino, CA, USA) with precision 0.1 cm and body weight with the same electronic scale (Seca Clara 803, Seca, Chino, CA, USA) with a precision of 0.1 kg, with the participant barefoot and in minimum clothing. BMI was calculated by dividing the individual's weight by height squared (kg/m^2^).

Body composition assessment was done using the Discovery A System (Hologic, Waltham, MA, USA), in accordance with published guidelines by the International Society for Clinical Densitometry [27]. Participants were placed centered on the scanning field in a supine position, with palms down and arms at sides, slightly separated from the trunk. Regions of interest (ROIs) were defined by the analytical program and included total body, trunk, head, pelvis, upper limbs, and lower limbs. For each region, DXA scanned weight of total, fat, and lean body mass. For the actual study, three lean mass measures were used: appendicular lean mass (ALM), calculated by the addition of the four limbs lean mass; ALM index (ALMI), computed as the ratio of ALM over height squared; and ALM divided by body mass index (ALM/BMI).

### Grip Strength (GS)

GS was assessed with a Baseline® hydraulic hand dynamometer (Fabrication Enterprises, Inc., White Plains, NY, USA), in the morning. According to the American Society of Hand Therapists guidelines [28], subjects were seated, shoulders adducted and neutrally rotated, elbow flexed at 90°, forearm in neutral position, and wrist between 0° and 30° of dorsiflexion. The highest value (expressed in kilograms) of three consecutive measurements with the self-reported dominant hand was used for the analysis.

### Sarcopenia Diagnosis

Classifications defined in Caucasian participants with cut-off values for lean mass measured by DXA and for muscle strength measured by GS were used (Table 1). We first classified participants based on the two current definitions. FNIH (Foundation for the National Institutes of Health Sarcopenia Project) sarcopenia diagnosis criteria (FNIH2014) identifies “severe sarcopenia” with ALM either in absolute values or adjusted to BMI [29]. The revised consensus definition of the European Working Group on Sarcopenia in Older People (EWGSOP2) [1] defines sarcopenia as “probable” in case of low GS, and “confirmed” if participants have both low GS and low muscle mass, either in absolute (ALM) or height-adjusted values (ALMI). *Probably sarcopenic* participants by EWGSOP2 criteria considers only low GS, they are thus not comparable to *sarcopenic* ones, which include low muscle mass, so they were added to the *non-sarcopenic*. Due to close or identical cut-off values for ALM in absolute values and GS, FNIH2014 and EWGSOP2 identified the same participants as *sarcopenic* and were considered together. Two other alternative definitions have also been applied: One using less stringent cut-offs to identify “mild to moderate sarcopenia” derived from part of the population of the original FNIH study (FNIH2017) [30] and the definition of the first EWGSOP consensus [31] in which participants are classified as *sarcopenic* in the presence of both low GS and low ALMI.

**Table 1:** Cut-off values of grip strength and lean mass assessed by DXA for sarcopenia diagnosis and classification in women, depending on the defined criteria

	Grip strength		Lean mass	
	Measure	Cut-point	Measure	Cut-point
FNIH 2014 [29]	Peak value (kg)	< 16.00	ALM (kg)	15.02
			ALM/BMI (kg/(kg/m^2^))	0.512
EWGSOP2, 2019 [1]	Peak value (kg)	< 16.00	ALM (kg)	15.00
			ALMI (kg/m^2^)	5.5
FNIH 2017 [30]	Peak value (kg)	< 19.99	ALM (kg)	14.12
			ALM/BMI (kg/(kg/m^2^))	0.591
EWGSOP, 2010 [31]	Peak value (kg)	< 20.00	ALMI (kg/m^2^)	5.5

*ALM* appendicular lean mass, *BMI* body mass index, *ALMI* ALM/height^2^, *FNIH* Foundation for the National Institutes of Health Sarcopenia Project; sarcopenia is defined by the presence of both low grip strength and low lean mass. 2014 criteria would define “severe sarcopenia”, while 2017 criteria would define “mild to moderate sarcopenia”, *EWGSOP2* European Working Group on Sarcopenia in Older People, revised consensus; sarcopenia is defined as “probable” in the presence of only low grip strength, and “confirmed” in the presence of both low grip strength and low lean mass, *EWGSOP* European Working Group on Sarcopenia in Older People; sarcopenia is defined by the presence of both low grip strength and low lean mass

### Statistical Analysis

Parameters were compared after classification of participants: (i) following different sarcopenia diagnosis criteria, (ii) by tertiles of sc-11AM and sc-8PM, and (iii) by tertiles of age.

Statistical analyses were conducted using Stata v16.1^©^ (StataCorp, College Station, TX, USA) for Windows^©^. Due to their skewed distributions, salivary cortisol values were log-transformed to approach a normal distribution. Descriptive results were expressed as number of participants (percentage) for categorical variables and as average ± standard deviation for continuous variables.

Between-group comparisons were performed using chi-square or Fisher’s exact test for categorical variables and Kruskal–Wallis test, student’s *t*-test, or analysis of variance for continuous variables. For continuous variables, multivariable analysis was conducted using analysis of variance adjusting for age and BMI; results were expressed as adjusted mean ± standard error. Associations between salivary cortisol markers and sarcopenia components were assessed using (1) Spearman correlation and (2) linear regression using sarcopenia components as dependent and cortisol markers as independent variables; results were expressed as slope, namely the regression coefficient, (standard error) for an increment of 10 nmol/l salivary cortisol. Statistical significance was considered for a two-sided test with *p*-value < 0.05.

## Results

### Selection of Participants

**Fig. 1:**
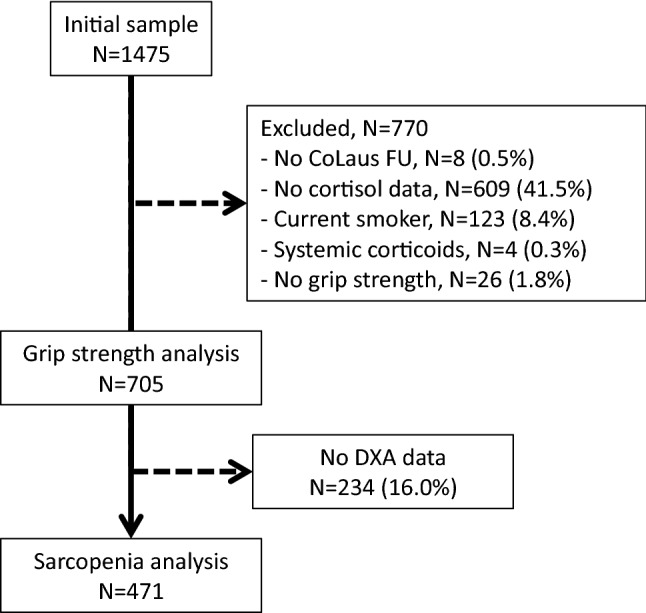
Flowchart of the study

Of the initial 1475 participants from OsteoLaus, 705 (47.8%) had both cortisol and GS measures (Fig. 1 and Supplementary Fig. 1) and 471 (31.9%) had cortisol, GS, and body composition measures (Fig. 1). Supplementary Table 1 summarizes the characteristics of included and excluded participants according to sarcopenia diagnosis. Included participants were younger, had higher GS measures, and lower sc-8PM levels.

### Participant’s Classification Using Different Criteria for Sarcopenia Diagnosis

Participants were classified as *sarcopenic* or *non-sarcopenic* depending on the cut-offs values in Table 1. As only EWGSOP2 criteria define *probably sarcopenic* by low GS alone, those were added to *non-sarcopenic* for the analysis. *Probably sarcopenic* by EWGSOP2-ALM (*n* = 21) were heavier (weight: 74.7 ± 12.3 vs. 67.7 ± 12.1 kg, *p* = 0.04; and BMI: 28.6 ± 4.7 vs. 25.9 ± 4.5 kg/m^2^, *p* = 0.02); and *probably sarcopenic* by EWGSOP2-ALMI (*n* = 33) were older (66.6 ± 7.4 vs. 62.6 ± 7.8 years, *p* = 0.012), shorter (158.6 ± 6.6 vs. 162.0 ± 6.7 cm, *p* = 0.02), and had lower ALM/BMI (0.62 ± 0.10 vs. 0.68 ± 0.10 kg/(kg/m^2^), *p* = 0.004), when compared with *non-sarcopenic*. There was no difference in any other measured parameter or any cortisol value (results not shown).

**Fig. 2:**
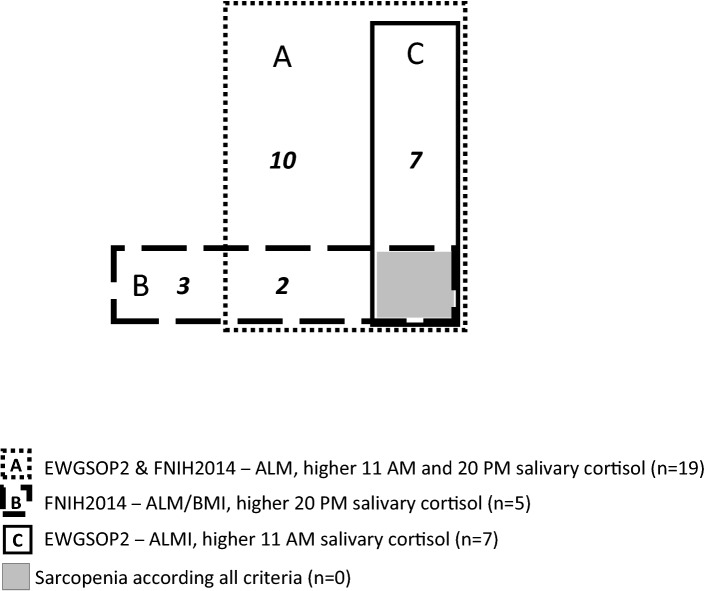
Number of participants diagnosed as sarcopenic depending on the current definitions, and their association with higher salivary cortisol at 11 AM, 20 PM, or both

The number of identified *sarcopenic* individuals is different depending on the criteria used, with almost no overlap, from *n* = 5 (1.1%) diagnosed by FNIH2014-ALM/BMI to *n* = 31 (6.6%) using the ALM/BMI cut-off. This resulted in a total of 50 *sarcopenic* participants when applying all definitions (10.6%) and 22 when applying the current definitions (Fig. 2); no participant is considered *sarcopenic* by all the 6 definitions (central gray square).

Characteristics of the participants are shown in Table 2a for current sarcopenia definitions and in Supplementary Table 2a for the alternative definitions. Participants identified as *sarcopenic* were older compared to *non-sarcopenic* across definitions. There were more sarcopenic participants in higher age tertiles (Supplementary Table 3), with a prevalence going from 0.0 to 1.9% in tertile 1 (50.3–59.0 years) to 1.9–12.1% in tertile 3 (66.5–80.8 years). Older participants had lower values of GS and ALM, either in absolute values or corrected to BMI or height (ALMI). Salivary cortisol values at 11 AM are significantly higher with age, with a similar trend for salivary cortisol values at 8 PM (Supplementary Table 3).

**Table 2:** Classification of participants according to current criteria for sarcopenia diagnosis

	FNIH 2014 & EWGSOP2-ALM			FNIH 2014-ALM/BMI			EWGSOP2-ALMI		
	None	Sarcopenic	*p*-value*	None	Sarcopenic	*p*-value*	None	Sarcopenic	*p*-value*
*(a) Participants’ characteristics*									
*N* (%)	452 (96.0)	19 (4.0)		466 (98.9)	5 (1.1)		464 (98.5)	7 (1.5)	
Age	62.7 ± 7.5	69.4 ± 6.6	< 0.001	63.0 ± 7.5	67.5 ± 10.6	0.18	62.9 ± 7.5	69.6 ± 3.7	0.02
Weight (kg)	68.0 ± 12.2	52.9 ± 7.6	< 0.001	67.5 ± 12.4	67.0 ± 11.1	0.93	67.8 ± 12.2	46.7 ± 3.5	< 0.001
Height (cm)	162.0 ± 6.6	155.7 ± 6.7	< 0.001	161.8 ± 6.7	151.3 ± 4.7	< 0.001	161.7 ± 6.8	159.8 ± 6.6	0.45
BMI (kg/m^2^)	26.0 ± 4.5	21.9 ± 3.4	< 0.001	25.8 ± 4.6	29.1 ± 3.2	0.11	25.9 ± 4.5	18.3 ± 0.6	< 0.001
ALM (kg)	17.4 ± 2.6	13.6 ± 1.0	NR	17.3 ± 2.6	14.6 ± 1.6	0.02	17.3 ± 2.6	12.9 ± 0.9	< 0.001
ALMI (kg/m^2^)	6.6 ± 0.9	5.6 ± 0.5	< 0.001	6.6 ± 0.9	6.3 ± 0.3	0.51	6.6 ± 0.9	5.0 ± 0.3	NR
ALM/BMI	0.68 ± 0.1	0.63 ± 0.08	0.03	0.68 ± 0.10	0.50 ± 0.01	NR	0.68 ± 0.10	0.71 ± 0.06	0.49
Grip strength (kg)	25.0 ± 5.4	13.9 ± 1.8	NR	24.7 ± 5.7	15.3 ± 1.0	NR	24.7 ± 5.6	13.4 ± 1.6	NR
*(b) Bivariate and multivariable analysis of salivary cortisol levels (nmol/l)*									
*N* (%)	452 (96.0)	19 (4.0)		466 (98.9)	5 (1.1)		464 (98.5)	7 (1.5)	
Awakening									
Bivariate	18.5 ± 9.0	21.5 ± 9.4	0.21	18.6 ± 9.0	19.9 ± 10.3	0.74	18.6 ± 9.0	21.4 ± 9.8	0.47
Multivariate	18.5 ± 0.4	21.3 ± 2.3	0.34	18.6 ± 0.4	20.0 ± 4.0	0.69	18.6 ± 0.4	21.0 ± 3.8	0.69
+ 30 min									
Bivariate	27.2 ± 11.9	29.4 ± 15.3	0.52	27.3 ± 12.0	23.8 ± 11.5	0.54	27.3 ± 12.1	26.5 ± 6.6	0.79
Multivariate	27.2 ± 0.6	29.6 ± 3.0	0.53	27.3 ± 0.6	24.4 ± 5.4	0.62	27.3 ± 0.6	26.0 ± 5.0	0.88
11 AM									
Bivariate	9.1 ± 4.5	12.6 ± 6.9	0.007	9.3 ± 4.7	8.8 ± 4.8	0.68	9.2 ± 4.5	16.9 ± 7.6	< 0.001
Multivariate	9.2 ± 0.2	11.9 ± 1.1	0.05	9.3 ± 0.2	8.7 ± 2.1	0.65	9.2 ± 0.2	15.9 ± 1.8	0.005
8 PM									
Bivariate	3.2 ± 1.9	5.1 ± 3.1	0.001	3.2 ± 1.9	6.2 ± 4.5	0.03	3.2 ± 2.0	4.5 ± 2.2	0.13
Multivariate	3.2 ± 0.1	5.1 ± 0.5	0.001	3.3 ± 0.1	6.1 ± 0.9	0.04	3.2 ± 0.1	4.6 ± 0.8	0.14
AUC									
Bivariate	4003 ± 1457	5047 ± 1450	0.006	4034 ± 1462	4689 ± 2060	0.54	4014 ± 1450	5866 ± 1674	0.006
Multivariate	4010 ± 74	4860 ± 387	0.03	4035 ± 74	4630 ± 656	0.60	4017 ± 73	5669 ± 608	0.02

Results are expressed as number (percentage), as mean ± standard deviation (Bivariate), or as age and body mass index-adjusted mean ± standard error (Multivariate). Between-group comparisons performed using student’s *t*-test in (a) and using analysis of variance in (b)

*N* number, *BMI* body mass index, *ALM* appendicular lean mass, *ALMI* ALM index (ALM/height^2^), *NR* not relevant (included in the definition of sarcopenia), *FNIH* Foundation for the National Institutes of Health Sarcopenia Project, *EWGSOP2* European Working Group on Sarcopenia in Older People, revised consensus, *AUC* salivary cortisol diurnal Area Under the Curve value

*p values were calculated on Log-transformed values

Anthropometric characteristics of participants identified as *sarcopenic* are different depending on the definition (Table 2a and Supplementary Table 2a). BMI is lower in *sarcopenic* than in *non-sarcopenic* participants for definitions using ALM or ALMI cut-offs, while it is higher as expected by definition when using ALM/BMI cut-offs. Moreover, when using ALM/BMI cut-offs ALMI is not different between *sarcopenic* and *non-sarcopenic* participants, and conversely when using ALMI cut-offs ALM/BMI is similar in both groups. Yet both are statistically lower in *sarcopenic* participants when absolute ALM cut-offs are used.

### Salivary Cortisol Values in *Sarcopenic* Participants

There was no difference in salivary cortisol between *sarcopenic* and *non-sarcopenic* participants at awakening, 30 min later (Table 2b and Supplementary Table 2b), or the CAR (data not shown). Sc-11AM was significantly higher in *sarcopenic* participants according to FNIH2014&EWGSOP2-ALM, EWGSOP2-ALMI, and EWGSOP-ALMI both in bivariate and multivariate analyses. Sc-8PM was significantly higher in *sarcopenic* participants according to FNIH2014&EWGSOP2-ALM, FNIH2014-ALM/BMI, and EWGSOP-ALMI both in bivariate and multivariate analyses. sc-AUC was higher in *sarcopenic* participants according to FNIH2014&EWGSOP2-ALM, EWGSOP2-ALMI, FNIH2017-ALM/BMI, and EWGSOP-ALMI, both in bivariate and multivariate analyses. When participants considered *sarcopenic* by any definition were analyzed, the three sc-11AM, sc-8PM, and sc-AUC were higher in *sarcopenic* participants in bivariate and multivariate analyses (Table 3). Figure 2 graphically shows which current sarcopenia definition is associated with higher sc-11AM, higher sc-8PM, or both.

**Table 3:** Characteristics of participants classified as *sarcopenic* by any definition

	Any sarcopenia definition		
	None	Sarcopenic	*p*-value
*N* (%)	421 (89.4)	50 (10.6)	
Age	62.6 ± 7.5	66.9 ± 7.1	< 0.001
Weight (kg)	68.2 ± 12.0	61.2 ± 13.4	< 0.001
Height (cm)	162.4 ± 6.5	155.5 ± 6.1	< 0.001
BMI (kg/m^2^)	25.9 ± 4.5	25.3 ± 5.2	0.41
ALM (kg)	17.6 ± 2.5	14.4 ± 2.0	NR
ALMI (kg/m^2^)	6.7 ± 0.9	6.0 ± 0.8	NR
ALM/BMI	0.69 ± 0.10	0.58 ± 0.08	NR
Grip strength (kg)	25.6 ± 5.1	15.8 ± 2.4	NR
Salivary cortisol (nmol/l)*			
Awakening			
Bivariate	18.6 ± 8.9	18.6 ± 9.9	0.98
Multivariable	18.6 ± 0.5	18.5 ± 1.4	0.88
+ 30 min			
Bivariate	27.3 ± 12.0	27.0 ± 12.2	0.95
Multivariable	27.3 ± 0.6	27.2 ± 1.9	0.89
11 AM			
Bivariate	9.0 ± 4.5	11.5 ± 5.6	0.003
Multivariable	9.1 ± 0.2	11.2 ± 0.7	0.01
8 PM			
Bivariate	3.2 ± 1.9	4.1 ± 2.5	0.01
Multivariable	3.2 ± 0.1	4.1 ± 0.3	0.03
AUC			
Bivariate	3964 ± 1440	4763 ± 1549	0.002
Multivariable	3974 ± 77	4670 ± 235	0.008

Results are expressed as mean ± standard deviation (Bivariate) or as age and body mass index-adjusted mean ± standard error (Multivariate). Between-group comparisons performed using student’s *t*-test or *analysis of variance

*N* number, *BMI* body mass index, *ALM* appendicular lean mass, *ALMI* ALM index (ALM/height^2^), *NR* not relevant (included in the definition of sarcopenia), *AUC* salivary cortisol diurnal Area Under the Curve value

*p values were calculated on Log-transformed values

### Participant’s Classification According to sc-11AM and sc-8PM Tertiles

Participants with higher sc-11AM (Table 4) were older, with a similar trend for those with higher sc-8PM (Table 4). There was no difference in any anthropometric or body composition parameter (weight, height, BMI, ALM, ALM/BMI, or ALMI) between cortisol tertiles. With increasing sc-11AM or sc-8PM, GS was lower, and there were more *sarcopenic* participants for all definitions except for FNIH2014-ALM/BMI (sc-11AM and sc-8PM) and FNIH2017-ALM (sc-11AM). These differences were statistically significant only for the identification of *sarcopenic* participants in sc-11AM tertiles classification with EWGSOP2-ALMI and FNIH2017-ALM/BMI criteria, and in sc-8PM tertiles classification with EWGSOP2&FNIH2014-ALM and FNIH2017-ALM criteria.

**Table 4:** Characteristics of the participants according to 11 AM or 8 PM cortisol tertiles

	11 AM cortisol tertiles				8 PM cortisol tertiles			
	Tertile 1	Tertile 2	Tertile 3	*p*-value	Tertile 1	Tertile 2	Tertile 3	*p*-value
Sample size	150	150	149		148	148	148	
Age (years)	61.2 ± 6.9	63.9 ± 7.9	64.0 ± 7.5	0.001	62.1 ± 7.0	63.1 ± 8.3	63.9 ± 7.3	0.13
Weight (kg)	67.7 ± 12.4	69.2 ± 12.7	65.3 ± 11.9	0.02	68.5 ± 12.8	66.4 ± 10.7	67.3 ± 13.0	0.35
Height (cm)	161.8 ± 7.1	162.1 ± 6.3	161.3 ± 6.6	0.57	162.2 ± 7.2	162.0 ± 6.4	160.9 ± 6.6	0.22
BMI (kg/m^2^)	25.9 ± 4.5	26.4 ± 4.6	25.2 ± 4.6	0.08	26.0 ± 4.5	25.4 ± 4.3	26.0 ± 4.7	0.42
ALM (kg)	17.4 ± 2.7	17.6 ± 2.7	16.8 ± 2.5	0.04	17.5 ± 2.8	17.2 ± 2.2	17.1 ± 2.9	0.49
ALMI (kg/m^2^)	6.6 ± 0.9	6.7 ± 0.9	6.5 ± 0.9	0.08	6.6 ± 0.9	6.6 ± 0.8	6.6 ± 0.9	0.81
ALM (kg)/BMI (kg/m^2^)	0.68 ± 0.10	0.68 ± 0.10	0.68 ± 0.10	0.80	0.68 ± 0.10	0.69 ± 0.10	0.67 ± 0.10	0.26
Grip strength (kg)	25.8 ± 5.4	24.3 ± 5.6	23.6 ± 6.0	0.005	25.1 ± 5.7	24.8 ± 5.8	23.8 ± 5.8	0.10
*Sarcopenia definition n (%)*^*#*^								
EWGSOP2 & FNIH2014-ALM	2 (1.3)	7 (4.7)	9 (6.0)	0.08	3 (2.0)	2 (1.4)	13 (8.8)	0.004
FNIH2014-ALM/BMI	1 (0.7)	3 (2.0)	1 (0.7)	0.63	0 (0)	2 (1.4)	3 (2.0)	0.38
EWGSOP2-ALMI	0 (0.0)	1 (0.7)	6 (4.0)	0.007	1 (0.7)	1 (0.7)	4 (2.7)	0.38
FNIH2017-ALM	6 (4.0)	9 (6.0)	8 (5.4)	0.75	7 (4.7)	3 (2.0)	13 (8.8)	0.04
FNIH2017-ALM/BMI	4 (2.7)	10 (6.7)	15 (10.1)	0.03	7 (4.7)	8 (5.4)	14 (9.5)	0.23
EWGSOP-ALMI	2 (1.3)	4 (2.7)	9 (6.0)	0.08	3 (2.0)	2 (1.4)	9 (6.1)	0.08
Any definition	7 (4.7)	16 (10.7)	24 (16.2)	0.004	12 (8.1)	10 (6.8)	25 (17.0)	0.01

Results are expressed as mean ± standard deviation or as number (percentage)

*BMI* body mass index, *ALM* appendicular lean mass, *ALMI* ALM index (ALM/height^2^), *FNIH* Foundation for the National Institutes of Health Sarcopenia Project, *EWGSOP* European Working Group on Sarcopenia in Older People

Between-group comparisons performed using analysis of variance or ^#^Fisher’s exact test

### Relationship Between Salivary Cortisol Values and Sarcopenia Components

GS was inversely related to sc-11AM (*r* = − 0.155), sc-8PM (*r* = − 0.124), and sc-AUC (*r* = − 0.158; all *p* ≤ 0.002) (Spearman correlation coefficients (Supplementary Table 4)); no relationship to salivary cortisol was observed for any ALM measure or any other parameter. The calculated correlation coefficient indicates that each 10 nmol/l increase of sc-11AM is associated with decrease of 1.758 (SE 0.472) kg of GS, and each 10 nmol/l increase sc-8PM is associated with decrease of 2.929 (SE 1.115) kg of GS.

## Discussion

In this postmenopausal women population-based study, sarcopenia is associated with higher salivary cortisol at 11 AM, 8 PM, and diurnal AUC. Our results suggest that increased cortisol exposure even at physiological levels may be associated to sarcopenia development. Moreover, there is a linear inverse relationship between both salivary cortisol values at 11 AM and 8 PM and grip strength.

### Sarcopenia Prevalence

Sarcopenia prevalence is low in our population, 1.1–6.6% depending on the definition used, 10.6% if all of the definitions are considered. Our rate is similar to that of some studies using DXA for muscle mass measures with or without GS (0.9–7.9% [3]), but is lower than reported in others (4.5–21.5% [32, 33]). The main difference is age, which is lower in our whole population (> 50 years versus > 65 years in the latter studies [32, 33]), but with higher proportion of sarcopenic participants in higher age tertiles. We cannot rule out higher sarcopenia prevalence in the whole cohort, as excluded participants were significantly older and had lower GS (Supplementary Table 1).

The different criteria used lead to the identification of different participants, as shown in Fig. 2, even when using the same consensus definition but with muscle mass considered as an absolute value (ALM), or corrected by BMI (ALM/BMI) or height (ALMI), as observed in previous studies [4, 6, 34]. Moreover, *sarcopenic* participants on ALM/BMI cut-offs do not have lower ALMI, and vice versa, and older and younger participants have similar BMI values, but lower ALM. BMI changes are probably reflecting fat mass changes in this population, and lower ALM/BMI values could identify some participants with only increased fat mass. The pertinence of one or the other adjustment is still debated [4, 6, 34].

### Salivary Cortisol Values in *Sarcopenic* Participants

Our study suggests that sarcopenia is associated to higher diurnal levels of salivary cortisol, and thus with blunted cortisol circadian rhythm: *sarcopenic* participants have higher salivary cortisol at 11 AM, 8 PM, and/or AUC values, and globally more *sarcopenic* participants are found in the highest tertile sc-11AM or sc-8PM. To our knowledge, this is the first study to show that this association is present regardless of the definition used, or considering all participants identified by any of them, which increases its significance.

### Relationship Between Salivary Cortisol Values and Sarcopenia Components

We found no significant correlation between the different salivary cortisol values and the ALM measures, eventually due to the exclusion of a potentially relevant number of sarcopenic participants as discussed before (Supplementary Table 1). Conversely, cortisol values were inversely associated to GS measures. Thus the relationship between salivary cortisol values and sarcopenia is mainly mediated by GS in our study. Interestingly, participants identified as *probably sarcopenic* by the presence of only a decreased GS (EWGSOP2 definitions) did not have higher cortisol levels at any time-point, even though *sarcopenic* diagnosis by EWGSOP2 cut-off values are the same. We hypothesize that participants with low GS and normal ALM (*probably sarcopenic*) represent a different population with lower performance of other origin. To our knowledge, whether this population evolves toward a sarcopenia diagnosis over time due to a decrease of ALM has never been studied.

Most published studies on cortisol and muscle quantity or function have not considered free cortisol (the active form measured in saliva or urines) or its circadian rhythm. Those who analyzed the relationship between free cortisol or circadian rhythm and DXA for ALM quantification, GS for muscle function assessment, or sarcopenia diagnosis using both methods [15, 16, 18, 19, 35, 36], have produced conflicting results. Two studies found a positive association between free cortisol and GS. Heaney et al. (36 healthy participants, 72.5 ± 6.5 years, 18 women) [36] found that lower overall salivary cortisol levels (driven by lower early morning and 30 min after waking values) were associated with lower GS. Older participants had higher mid-day values, but were not separately analyzed. In another study with 798 all-age participants (mean age 48.6 ± 17.3 years, 45.1% women) [35], Bochud et al. showed a positive association between GS and total lean mass/height^2^ (but not ALMI) measured by DXA, and different cortisol and cortisone metabolites measured in urine; the association was not present in older participants, and the authors did not specifically analyze free cortisol. Two other studies found lower muscle function with higher evening salivary cortisol, but no relationship with GS in particular in the cross-sectional analysis [18]. In the first study (1046 participants, mean age 74.5 ± 7.0 years, 51.8% women) [16], during a 3-year follow-up GS loss was higher in participants with higher evening salivary cortisol values. In the second study, an individual meta-analysis of cortisol and muscle function data from several cohorts (participants mean age of 61–74 years) [18], the authors found no association between salivary cortisol and GS measures. However, only two cohorts had both measures, one with non-published data, and the previously discussed negative study [16], and higher salivary cortisol levels were associated with poorer performance in other tests of muscle function (walking speed, chair rises and standing balance) [17].

Finally, two studies showed a relationship between higher salivary cortisol and sarcopenia or muscle function [19]. In one small study (*n* = 45, mean age 77.6 ± 6.5 years, 21 women) [19] *sarcopenic* participants as defined by Baumgartner et al. [37] (on the sole basis of ALMI, with similar cut-off values as EWGSOP-ALMI definition) had higher diurnal sc-AUC than *sarcopenic obese*, *obese*, and *normal* patients. In a population-study including 745 participants (mean age 75.1 years, range 65–90, 363 women) [15] frail and prefrail patients had higher evening cortisol than nonfrail, which was associated to low GS and slow gait speed.

All described studies included both men and women, and either found no gender difference [16] or did not analyze data separately, which may have increased the variance of the GS or ALM measures, since men have higher values. In addition, there is great heterogeneity between studies in the definition of normal cortisol values: some used a cut-off (20% of the population with lower values [15] or different published criteria [19, 36]); others looked for linear associations of either the *z*-score [18] or absolute values [35]; one study compared values between participants with highest or lower salivary cortisol [16].

### Strengths and Limitations

Main limitations of our study are due to the cross-sectional observational design. Also, we only collected one salivary cortisol probe per time-point per participant, and evening values have not been measured at midnight, as proposed for hypercortisolism screening, to facilitate compliance to saliva collection. Muscle function displays significant circadian variations [38], and GS has been measured in the morning but not at the same hour in every participant, and salivary cortisol and GS have not been measured the same day. Finally, awakening hour has not been recorded. Exclusion of participants decreases sample size and limits the generalization. However included participants had higher GS measures and had lower evening cortisol; it is probable that data of the full cohort would have yielded similar results.

On the other hand, the study has multiple strengths. The OsteoLaus cohort is a large homogeneous population sample of postmenopausal women that allows for adequate statistical power. The most accurate method for body composition assessment, DXA, has been used. Also, even though multiple association analysis have been made to characterize the population, results are similar and highly significant whatever sarcopenia definition is used, and for both 11 AM and 8 PM time-points, and the resulting sc-AUC values.

## Conclusion

Our study suggests that increased diurnal cortisol impregnation with aging is associated to sarcopenia diagnosis in postmenopausal women, and may have a role in its development. More studies are needed, mainly with longitudinal analysis, to determine whether salivary cortisol, an easy and non-stressful method of cortisol measurement, could have a clinical utility in identifying patients at risk for sarcopenia.

## Supplementary Information

Below is the link to the electronic supplementary material.

Supplementary file1 (DOCX 78 kb)

## Data Availability

The datasets generated during and/or analyzed during the current study are not publicly available but are available from the corresponding author on reasonable request.
