# Supplementary material for: Diurnal Salivary Cortisol in Sarcopenic Postmenopausal Women: The OsteoLaus Cohort
Source: Calcif Tissue Int. 2021 May 18;109(5):499–509. doi: 10.1007/s00223-021-00863-y (PMC8484096; doi:10.1007/s00223-021-00863-y)
Supplement: Supplementary file 1 — Supplementary file1 (DOCX 78 kb) [file 223_2021_863_MOESM1_ESM.docx]

**Supplementary information**

Diurnal salivary cortisol in sarcopenic postmenopausal women: the OsteoLaus cohort

Osteoporosis International

Elena Gonzalez Rodriguez^1^; Pedro Marques-Vidal; Bérengère Aubry-Rozier; Georgios Papadakis; Martin Preisig; Christine Kuehner; Peter Vollenweider; Gerard Waeber; Didier Hans and Olivier Lamy

Affiliations

1 Interdisciplinary Center for Bone Diseases, Service of Rhumatology, Lausanne University Hospital and University of Lausanne, Switzerland;

[elena.gonzalez-rodriguez@chuv.ch](mailto:elena.gonzalez-rodriguez@chuv.ch)

**Supplementary table 1:** Comparison between participants included for sarcopenia diagnosis, and excluded participants

|  | **All** | **Included** | **Excluded** | **p-value** |
| --- | --- | --- | --- | --- |
| Sample size | 1467 | 471 | 996 |  |
| Age (years) | 64.5 ± 7.6 | 63.0 ± 7.5 | 65.2 ± 7.5 | *<0.001* |
| Weight (kg) | 67.3 ± 12.1 | 67.5 ± 12.4 | 67.3 ± 12.0 | 0.78 |
| Height (cm) | 161.3 ± 6.7 | 161.7 ± 6.8 | 161.1 ± 6.6 | 0.10 |
| BMI (kg/m^2^) | 25.9 ± 4.5 | 25.8 ± 4.5 | 26.0 ± 4.5 | 0.59 |
| ALM (kg) | 17.1 ± 2.6 | 17.3 ± 2.6 | 17.0 ± 2.5 | 0.08 |
| ALMI (kg/m^2^) | 6.6 ± 0.8 | 6.6 ± 0.9 | 6.5 ± 0.8 | 0.16 |
| ALM (Kg)/BMI (Kg/m^2^) | 0.68 ± 0.10 | 0.68 ± 0.10 | 0.68 ± 0.11 | 0.76 |
| Grip strength (kg) | 23.9 ± 5.9 | 24.6 ± 5.7 | 23.6 ± 6.0 | *0.005* |
| Salivary cortisol (nmol/l)* |  |  |  |  |
| Awakening | 18.5 ± 9.2 | 18.6 ± 9.0 | 18.4 ± 9.4 | 0.79 |
| + 30 min | 27.0 ± 11.9 | 27.4 ± 12.2 | 26.6 ± 11.6 | 0.52 |
| 11 AM | 9.6 ± 5.8 | 9.3 ± 4.7 | 10.0 ± 7.0 | 0.26 |
| 8 PM | 3.6 ± 3.2 | 3.3 ± 2.3 | 3.9 ± 4.0 | *0.003* |
| AUC | 4262 ± 2152 | 4066 ± 1530 | 4517 ± 2742 | *0.009* |

Exclusion criteria: no CoLaus data, no cortisol data, current smoker, and absence of Grip strength or DXA data. BMI: body mass index. ALM: appendicular lean mass. ALMI: ALM index (ALM/height^2^). AUC: salivary cortisol diurnal Area Under the Curve value. Results are expressed as mean ± standard deviation. Between-group comparisons performed using student’s t-test or *Kruskal-Wallis test.

**Supplementary table 2a**: Classification of participants according to alternative criteria for sarcopenia diagnosis.

**a:** Participants’ characteristics.

|  | **FINH 2017 – ALM** | | | **FNIH 2017 – ALM/BMI** | | | **EWGSOP – ALMI** | | |
| --- | --- | --- | --- | --- | --- | --- | --- | --- | --- |
|  | **None** | **Sarcopenic** | **p-value** | **None** | **Sarcopenic** | **p-value** | **None** | **Sarcopenic** | **p-value** |
| N (%) | 446 (94.7) | 25 (5.3) |  | 440 (93.4) | 31 (6.6) |  | 456 (96.8) | 15 (3.2) |  |
| Age | 62.8 ± 7.5 | 67.0 ± 6.6 | 0.006 | 62.7 ± 7.5 | 67.2 ± 6.8 | 0.001 | 62.9 ± 7.5 | 67.2 ± 5.7 | 0.03 |
| Weight (kg) | 68.3 ± 12.1 | 53.0 ± 7.8 | <0.001 | 67.4 ± 12.4 | 68.0 ± 12.3 | 0.80 | 68.0 ± 12.1 | 51.6 ± 8.1 | <0.001 |
| Height (cm) | 162.1 ± 6.5 | 154.1 ± 6.5 | <0.001 | 162.2 ± 6.5 | 154.2 ± 5.6 | <0.001 | 161.8 ± 6.7 | 158.4 ± 7.1 | 0.05 |
| BMI (Kg/m^2^) | 26.0 ± 4.5 | 22.4 ± 3.5 | <0.001 | 25.6 ± 4.5 | 28.4 ± 3.9 | <0.001 | 26.0 ± 4.5 | 20.6 ± 3.1 | <0.001 |
| ALM (kg) | 17.5 ± 2.5 | 13.0 ± 0.7 | NR | 17.4 ± 2.6 | 15.1 ± 2.2 | <0.001 | 17.4 ± 2.6 | 13.0 ± 0.9 | <0.001 |
| ALMI (kg/m^2^) | 6.7 ± 0.8 | 5.5 ± 0.4 | <0.001 | 6.6 ± 0.9 | 6.3 ± 0.7 | 0.08 | 6.6 ± 0.8 | 5.2 ± 0.3 | NR |
| ALM/BMI | 0.68 ± 0.10 | 0.59 ± 0.09 | <0.001 | 0.69 ± 0.10 | 0.53 ± 0.04 | NR | 0.68 ± 0.10 | 0.64 ± 0.10 | 0.18 |
| Grip strength (kg) | 25.0 ± 5.4 | 16.1 ± 2.6 | NR | 25.2 ± 5.4 | 16.1 ± 2.3 | NR | 24.8 ± 5.6 | 15.9 ± 2.8 | NR |

**b**: Bivariate and multivariable analysis of salivary cortisol levels (nmol/l).

|  | **FINH 2017 – ALM** | | | **FNIH 2017 – ALM/BMI** | | | **EWGSOP – ALMI** | | |
| --- | --- | --- | --- | --- | --- | --- | --- | --- | --- |
|  | **None** | **Sarcopenic** | **p-value*** | **None** | **Sarcopenic** | **p-value*** | **None** | **Sarcopenic** | **p-value*** |
| N (%) | 446 (94.7) | 25 (5.3) |  | 440 (93.4) | 31 (6.6) |  | 456 (96.8) | 15 (3.2) |  |
| Awakening |  |  |  |  |  |  |  |  |  |
| Bivariate | 18.5 ± 9.0 | 19.9 ± 8.3 | 0.30 | 18.6 ± 8.8 | 18.8 ± 11.6 | 0.83 | 18.7 ± 9.0 | 17.0 ± 7.6 | 0.79 |
| Multivariable | 18.6 ± 0.4 | 19.8 ± 2.0 | 0.47 | 18.6 ± 0.4 | 18.9 ± 1.8 | 0.91 | 18.7 ± 0.4 | 16.6 ± 2.6 | 0.52 |
| +30 min |  |  |  |  |  |  |  |  |  |
| Bivariate | 27.2 ± 11.9 | 29.3 ± 14.8 | 0.47 | 27.3 ± 11.9 | 27.1 ± 13.3 | 0.93 | 27.3 ± 12.1 | 25.2 ± 6.0 | 0.98 |
| Multivariable | 27.2 ± 0.6 | 29.2 ± 2.7 | 0.54 | 27.2 ± 0.6 | 27.8 ± 2.4 | 0.87 | 27.3 ± 0.6 | 24.7 ± 3.4 | 0.91 |
| 11 AM |  |  |  |  |  |  |  |  |  |
| Bivariate | 9.2 ± 4.6 | 10.3 ± 6.3 | 0.65 | 9.2 ± 4.7 | 10.7 ± 4.6 | 0.13 | 9.1 ± 4.6 | 13.2 ± 6.5 | ***0.003*** |
| Multivariable | 9.3 ± 0.2 | 9.6 ± 1.0 | 0.79 | 9.2 ± 0.2 | 10.8 ± 0.9 | 0.15 | 9.2 ± 0.2 | 12.5 ± 1.2 | ***0.02*** |
| 8 PM |  |  |  |  |  |  |  |  |  |
| Bivariate | 3.2 ± 1.9 | 4.5 ± 3.1 | 0.08 | 3.2 ± 1.9 | 4.1 ± 2.8 | 0.13 | 3.2 ± 2.0 | 4.2 ± 1.8 | ***0.03*** |
| Multivariable | 3.2 ± 0.1 | 4.5 ± 0.4 | 0.09 | 3.2 ± 0.1 | 4.0 ± 0.4 | 0.22 | 3.2 ± 0.1 | 4.3 ± 0.5 | ***0.04*** |
| AUC |  |  |  |  |  |  |  |  |  |
| Bivariate | 4013 ± 1459 | 4599 ± 1586 | 0.09 | 3998 ± 1458 | 4758 ± 1497 | ***0.02*** | 4007 ± 1455 | 5073 ± 1558 | ***0.01*** |
| Multivariable | 4019 ± 75 | 4476 ± 335 | 0.20 | 4001 ± 75 | 4717 ± 308 | ***0.03*** | 4012 ± 74 | 4933 ± 416 | ***0.04*** |

N: number. BMI: body mass index. ALM: appendicular lean mass. ALMI: ALM index (ALM/height^2^). NR: not relevant (included in the definition of sarcopenia). FNIH: Foundation for the National Institutes of Health Sarcopenia Project. EWGSOP: European Working Group on Sarcopenia in Older People. AUC: salivary cortisol diurnal Area Under the Curve value. Results are expressed as number (percentage), as mean ± standard deviation (Bivariate), or as age and body mass index-adjusted mean ± standard error (Multivariate). Between-group comparisons performed using student’s t-test in (a), and using analysis of variance in (b). *p-values were calculated on Log transformed values.

**Supplementary table 3**: Characteristics of the participants according to age tertiles

|  | **Tertile 1** | **Tertile 2** | **Tertile 3** | **p-value** | **p-value**  **for trend** |
| --- | --- | --- | --- | --- | --- |
| Sample size | 159 | 155 | 157 |  |  |
| Age (years) | 50.3-59.0 | 59.1-66.4 | 66.5-80.8 | - | - |
| Weight (kg) | 68.0 ± 12.4 | 67.2 ± 12.0 | 67.2 ± 12.7 | 0.82 | 0.60 |
| Height (cm) | 162.3 ± 7.0 | 162.3 ± 6.6 | 160.5 ± 6.5 | *0.02* | *0.01* |
| BMI (kg/m^2^) | 25.8 ± 4.6 | 25.5 ± 4.5 | 26.1 ± 4.6 | 0.54 | 0.57 |
| Grip strength (Kg) | 26.6 ± 5.8 | 25.4 ± 5.0 | 21.7 ± 5.1 | *<0.001* | *<0.001* |
| ALM (Kg) | 18.0 ± 2.7 | 17.2 ± 2.5 | 16.7 ± 2.5 | *<0.001* | *<0.001* |
| ALMI (Kg/m^2^) | 6.8 ± 0.9 | 6.5 ± 0.8 | 6.5 ± 0.9 | *<0.001* | *<0.001* |
| ALM (Kg)/BMI (Kg/m^2^) | 0.71 ± 0.11 | 0.68 ± 0.10 | 0.65 ± 0.09 | *<0.001* | *<0.001* |
| Sarcopenia definition n (%)^#^ |  |  |  |  |  |
| EWGSOP2 & FNIH2014 – ALM | 1 (0.6) | 4 (2.6) | 14 (8.9) | *<0.001* |  |
| FNIH2014 – ALM/BMI | 1 (0.6) | 1 (0.6) | 3 (1.9) | 0.54 |  |
| EWGSOP2/ALMI | 0 (0.0) | 1 (0.7) | 6 (3.8) | *0.01* |  |
| FNIH2017 – ALM | 2 (1.3) | 7 (4.5) | 16 (10.2) | *0.002* |  |
| FNIH2017 – ALM/BMI | 3 (1.9) | 9 (5.8) | 19 (12.1) | *0.001* |  |
| EWGSOP – ALMI | 1 (0.6) | 4 (2.6) | 10 (6.4) | *0.01* |  |
| Any definition | 6 (3.8) | 13 (8.4) | 31 (19.8) | *<0.001* |  |
| Salivary cortisol (nmol/l)* |  |  |  |  |  |
| Awakening | 19.3 ± 9.4 | 17.8 ± 8.5 | 18.8 ± 9.1 | 0.61 | 0.84 |
| + 30 min | 27.8 ± 11.8 | 28.0 ± 12.8 | 26.0 ± 11.3 | 0.31 | 0.16 |
| 11 AM | 8.7 ± 4.3 | 9.2 ± 4.7 | 10.0 ± 5.0 | 0.05 | *0.02* |
| 8 PM | 3.1 ± 1.8 | 3.2 ± 2.0 | 3.5 ± 2.1 | 0.11 | 0.07 |
| AUC | 3861 ± 1324 | 3989 ± 1517 | 4281 ± 1538 | 0.05 | *0.02* |

BMI: body mass index. ALM: appendicular lean mass. ALMI: ALM index (ALM/height^2^). AUC: Salivary cortisol diurnal Area Under the Curve value. FNIH: Foundation for the National Institutes of Health Sarcopenia Project. EWGSOP: European Working Group on Sarcopenia in Older People. Results are expressed as mean ± standard deviation or n (percentage). Between-group comparisons performed using analysis of variance or ^#^Fisher’s exact test. *p-values were calculated on Log transformed values.

**Supplementary table 4**: Spearman correlations between salivary cortisol markers and components of sarcopenia.

|  | **ALM** | **ALM/BMI** | **ALMI** | **Grip strength** |
| --- | --- | --- | --- | --- |
| Awakening |  |  |  |  |
| Spearman r | -0.074 | -0.016 | -0.029 | -0.053 |
| P-value | 0.122 | 0.738 | 0.553 | 0.178 |
| N | 434 | 434 | 434 | 644 |
| +30 min |  |  |  |  |
| Spearman r | -0.012 | 0.035 | -0.011 | -0.005 |
| P-value | 0.804 | 0.462 | 0.821 | 0.896 |
| N | 435 | 435 | 435 | 650 |
| 11 AM |  |  |  |  |
| Spearman r | -0.092 | 0.010 | -0.064 | -0.153 |
| P-value | 0.052 | 0.834 | 0.176 | ***<0.001*** |
| N | 449 | 449 | 449 | 671 |
| 8 PM |  |  |  |  |
| Spearman r | -0.055 | -0.054 | -0.005 | -0.118 |
| P-value | 0.249 | 0.259 | 0.914 | ***0.002*** |
| N | 444 | 444 | 444 | 663 |
| AUC |  |  |  |  |
| Spearman r | -0.071 | 0.000 | -0.018 | -0.155 |
| P-value | 0.159 | 0.998 | 0.723 | ***<0.001*** |
| N | 397 | 397 | 397 | 588 |

ALM: appendicular lean mass. BMI: body mass index. ALMI: ALM index (ALM/height^2^). N: number. AUC: salivary cortisol diurnal Area Under the Curve value. Results are expressed as Spearman correlation and p-value.

**Supplementary figure 1:** Sampling procedure.

CoLaus

**Baseline: 2009-2012**

**n=1475 ♀**

OsteoLaus

**1^st^ follow-up: 2009-2013**

**n=5064**

Frailty substudy

**n=3704**

**n=705**

PsyCoLaus

**1^st^ follow-up: 2009-2013**

**n=4004**

*CoLaus/PsyCoLaus*: population-based cohort of randomly selected Caucasians living in the City of Lausanne. Collected data: treatment history data, measures of salivary cortisol circadian rhythm. *Frailty substudy*: measures of peak grip strength (GS). *OsteoLaus*: women >50 years old from CoLaus study. Collected data: age at examination, antropometric measures, body composition assessment by dual X-rax absorptiometry (DXA).
